# NeRF-Based Three-Dimensional Reconstruction for Large-Diameter Rescue Shafts

**DOI:** 10.3390/s26123847

**Published:** 2026-06-17

**Authors:** Hairong Gu, Jiaxi Wang, Chenggang Chen, Wenjuan Yang, Mostak Ahamed, Zujie Zou

**Affiliations:** 1 National Engineering Research Center of Highway Maintenance Technology, Chang’an University, Xi’an 710064, China; guhairong@chd.edu.cn (H.G.); 2024125079@chd.edu.cn (J.W.); mostakahamed002@gmail.com (M.A.); 2 Anhui Key Laboratory of Intelligent Manufacturing of Construction Machinery, Anhui Jianzhu University, Hefei 230601, China; 3 Guangxi Mechanical Industry Research Institute, Nanning 530007, China; 4 School of Water and Environment, Chang’an University, Xi’an 710064, China; ywj_xa@chd.edu.cn; 5 Xi’an Research Institute of China Coal Technology and Engineering Group Corporation, Xi’an 710077, China; zouzujie@cctegxian.com

**Keywords:** neural radiance fields (NeRF), 3D reconstruction, large-diameter rescue shafts, NR-SNR

## Abstract

Large-diameter rescue shafts serve as critical infrastructure for emergency response in mining disaster scenarios, and their structural deformation directly affects the safe passage of rescue capsules. In this paper, we investigate three-dimensional (3D) reconstruction techniques for large-diameter rescue shaft environments and develop a Neural Radiance Fields (NeRF)-based reconstruction and deformation assessment scheme. The proposed workflow integrates no reference signal-to-noise-ratio (NR-SNR), image-quality filtering, SfM-based camera-pose estimation, Nerfacto reconstruction, point-cloud export, and circular-section fitting. The NR-SNR retention-ratio experiment shows that retaining approximately 35% high-quality images provides a practical efficiency–quality trade-off for the present dataset, reducing the computational burden of SfM pose estimation while preserving sufficient geometric information for subsequent reconstruction. The reconstructed radiance field is further exported as a dense point cloud and evaluated using relative radius error, circle-fitting residuals, and image-level rendering metrics. Experiments on a simulated large-diameter rescue shaft platform show that the proposed NeRF-based scheme provides favorable geometric measurement applicability and visual reconstruction quality under weak-texture and low-illumination conditions. Compared with conventional MVS and the tested 3DGS baseline, the proposed scheme produces a point-cloud output that is more suitable for subsequent circular-section fitting and deformation-related assessment. In addition, comparison with a representative SDF-based baseline indicates that direct implicit surface recovery remains challenging for the tested hollow cylindrical shaft-wall scene. The results demonstrate the potential of the proposed NeRF-based workflow for rescue-shaft inner-wall reconstruction and engineering-oriented deformation evaluation.

## 1. Introduction

Large-diameter rescue shafts play an important role in emergency mine rescue operations, and their structural integrity and deformation characteristics directly affect the safe passage of rescue capsules. As mining depth increases, the surrounding geomechanical environment becomes more complex, potentially aggravating shaft-wall deformation, radial shrinkage, inclination, and local obstruction. Therefore, efficient and geometrically usable three-dimensional (3D) reconstruction of the inner wall of large-diameter rescue shafts is essential for deformation assessment and passability evaluation during rescue operations [1].

In recent years, 3D reconstruction has been widely used to recover real-world objects or scenes from two-dimensional (2D) images or sensor measurements into digital 3D models. It enables the acquisition of dimensional, volumetric, and shape information, and provides a digital basis for engineering inspection and structural evaluation. Several approaches have been proposed in the literature to detect shaft or shaft-like structural deformation. Contact-type multi-arm calipers can directly measure borehole or shaft profiles, but their accuracy is closely related to the density and arrangement of the measuring arms [2]. Laser- and LiDAR-based systems have also been widely investigated for shaft-wall contour extraction, inclination monitoring, and underground mapping [3,4,5,6,7]. For example, Vrochidis et al. developed an automatic elevator-shaft inspection system using computer vision and optical sensors, where a low-cost multi-sensor device integrating a Jetson Nano, LiDAR, laser sensors, and an MPU was used to scan the shaft and evaluate its dimensions [4]. Similar studies have also explored consumer-grade LiDAR, camera-LiDAR systems, SLAM-based monitoring, and underground LiDAR odometry for mineshaft, vertical-shaft, pipe, and confined-space inspection [3,5,6,7,8]. These studies demonstrate the feasibility of 3D inspection in shaft-like environments. However, such systems may still be limited by device deployment, calibration, occlusion, equipment size, cost, and operational constraints in narrow or emergency environments.

Vision-based structure-from-motion and multi-view stereo (SfM-MVS) methods provide a lightweight and low-cost alternative for 3D reconstruction [9,10]. By exploiting geometric relationships across multiple views, MVS can recover detailed 3D models when sufficient texture, stable illumination, and reliable feature correspondences are available. Nevertheless, rescue shafts usually exhibit weak illumination, narrow imaging spaces, repetitive cylindrical structures, and large low-texture wall regions. These conditions reduce the number and quality of feature matches and may lead to incomplete camera registration, sparse point clouds, holes, or noisy geometry. Recent point-cloud optimization studies have also shown that photogrammetric point clouds are affected by acquisition and processing parameters such as reprojection error, projection accuracy, intersection angle, camera–point distance, and image redundancy [11,12]. Therefore, image quality control and reconstruction reliability analysis are necessary when applying image-based reconstruction methods to engineering measurement tasks. Similar engineering reconstruction studies have also emphasized that reconstructed point clouds should be further connected with task-specific geometric quantities, such as volume, dimension, or deformation indicators, rather than being evaluated only from a visual perspective [13]. In addition, measurement-oriented sensing studies have highlighted the importance of uncertainty analysis and metrological validation when sensor outputs are used for quantitative engineering assessment [14].

More recently, radiance-field-based reconstruction methods have provided new possibilities for 3D modeling from posed images. Three-dimensional Gaussian Splatting (3DGS), as an explicit radiance-field representation, has demonstrated high rendering efficiency and strong visual performance [15]. However, the standard 3DGS pipeline may be sensitive to the quality of SfM initialization, especially in weak-texture environments where sparse point clouds are incomplete or inaccurate. Recent variants have attempted to relax this dependence on accurate initialization, indicating the rapid development of Gaussian-based reconstruction methods [16,17]. In parallel, neural radiance fields (NeRFs) represent scene geometry and appearance using a continuous implicit field and optimize the scene through differentiable volume rendering [18]. Compared with MVS, NeRF-based methods are less directly dependent on dense local feature correspondences and have shown potential in scenarios involving sparse viewpoints, weak textures, and complex lighting conditions [19,20,21,22,23,24].

Apart from NeRF variants based on density representation, SDF-based neural surface reconstruction methods, such as NeuS, VolSDF, Neuralangelo, and their follow-up variants, have been developed to recover more explicit surfaces with stronger geometric constraints [25,26,27,28]. Owing to their surface priors, these methods are often effective for reconstructing closed or watertight surfaces in common object-centric datasets. However, large-diameter rescue shafts constitute a different reconstruction scenario. The target surface is an elongated hollow cylindrical inner wall with monotonous and low-contrast textures. Meanwhile, the camera trajectory is constrained by the shaft geometry, and reliable ground-truth geometric data are difficult to acquire. Therefore, candidate reconstruction schemes should not be evaluated only by their theoretical surface modeling ability, but also by whether the reconstructed geometry can support subsequent engineering measurement workflows.

For this reason, this study adopts Nerfacto as the reconstruction backbone and builds a rescue-shaft-oriented reconstruction–measurement workflow around it. Nerfacto is implemented in the Nerfstudio framework and combines several practical developments from recent NeRF studies, including multi-resolution hash encoding, proposal-based sampling, appearance conditioning, and camera-pose refinement [19,20,21].

These components provide a balanced implementation for real-scene radiance-field reconstruction in terms of training efficiency, rendering quality, and practical usability. For the rescue-shaft scenario considered in this study, the key objective is not limited to novel-view synthesis. A more important requirement is whether the reconstructed radiance field can be exported as a point cloud and further used for circular-section fitting, radius estimation, deformation analysis, and passability evaluation. Existing radiance-field studies mainly focus on general indoor and outdoor scenes, object-level reconstruction, or rendering quality, while hollow cylindrical rescue-shaft inner-wall reconstruction and radius-based deformation assessment remain insufficiently investigated.

Therefore, this paper proposes a NeRF-based 3D reconstruction and deformation assessment scheme for large-diameter rescue shafts. The proposed scheme organizes image-quality filtering, SfM-based camera-pose estimation, Nerfacto reconstruction, point-cloud export, circular-section fitting, and passability evaluation into an engineering-oriented pipeline. The study aims to examine whether such a pipeline can provide a practical solution for low-texture rescue-shaft reconstruction, and to offer comparative evidence in terms of reconstruction quality, geometric usability, and computational cost.

The main contributions of this study are summarized as follows.

(1) A task-oriented NeRF-based reconstruction and measurement framework is established for large-diameter rescue shafts under weak-illumination and low-texture conditions. Unlike a purely visual reconstruction workflow, the proposed framework links neural reconstruction results with shaft-wall geometric measurement and rescue-capsule passability assessment.
(2) The influence of NR-SNR image filtering on reconstruction performance is investigated using different image-retention ratios. The selected 35% retention ratio is interpreted as a dataset-specific efficiency–quality trade-off rather than a universal threshold.
(3) The proposed scheme is evaluated from both rendering and engineering-measurement perspectives. In addition to PSNR, SSIM, and LPIPS, radius fitting, physical-diameter-based error, and circle-fitting residuals are used to assess the geometric applicability of the reconstructed shaft-wall point cloud.
(4) Comparative experiments and computational analyses are provided to support the engineering evaluation of the proposed workflow. The comparison includes conventional MVS, 3DGS, and a representative SDF-based baseline, while the computational analysis reports training iterations, learning-rate settings, ray batch size, hardware configuration, runtime, GPU memory consumption, and point-cloud export settings. The limitations of the workflow under low-texture, reflective, dynamic, and non-cylindrical deformation conditions are further discussed.

## 2. Materials and Methods

### 2.1. Principle of NeRF

**Figure 1:**
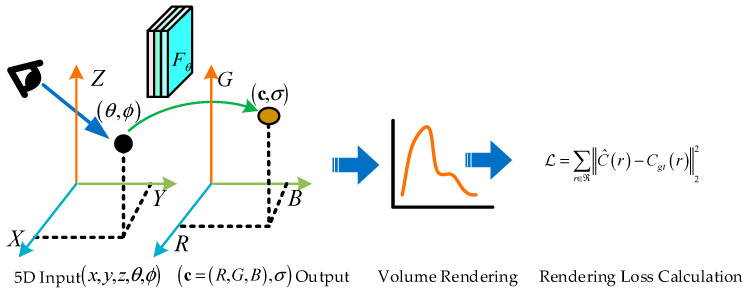
Schematic of the NeRF framework. A 5D input $\left(x,y,z,\theta ,\phi \right)$, consisting of a 3D spatial position and a 2D viewing direction, is fed into the radiance field $F_{\theta }$, which outputs the corresponding color $c=\left(R,G,B\right)$ and volume density σ. These outputs are then used in volume rendering and rendering loss calculation. The arrows indicate the directional flow of the process, and the different colors are used to distinguish the main components in the pipeline.

It is well known that NeRF is a novel 3D reconstruction technique that utilizes implicit representation, as illustrated in Figure 1. By taking a 3D spatial location and a 2D viewing direction as input, it models the scene using a fully connected deep neural network, specifically a multi-layer perceptron (MLP). It predicts the corresponding color $\left(R,G,B\right)$ and volume density σ.

The radiance field function can be formulated as

(1) $$F_{\theta }:\left(x,y,z,\theta ,\phi \right)\to \left(c=\left(R,G,B\right),\sigma \right)$$

where $\left(x,\;y,\;z\right)$ denotes the 3D spatial position of a sampled point, and (*θ*, *φ*) denotes the viewing direction. The output $c=\left(R,G,B\right)$ represents the predicted RGB color, and σ denotes the volume density at the corresponding spatial position and viewing direction.

Subsequently, NeRF utilizes classical volume rendering theory [29] to conduct front-to-back accumulation of the colors and densities predicted by the MLP, thereby deriving the final pixel color for each ray $\hat{C}\left(r\right)$, which can be expressed as

(2) $$\hat{C}\left(r\right)=\sum_{i=1}^{N}\exp\left(-\sum_{j=1}^{i-1}\sigma_{j}\delta_{j}\left(1-\exp\left(-\sigma_{i}\delta_{i}\right)\right)c_{i}\right)$$

where r denotes a camera ray, N is the number of sampled points along the ray, and $c_{i}$ and $\sigma_{i}$ represent the predicted color and volume density of the i-th sampled point, respectively. $\delta_{j}$ denotes the distance interval between adjacent sampled points. The exponential term describes the accumulated transmittance from the ray origin to the i-th sampled point.

Finally, NeRF computes a pixel-wise loss between the rendered and ground-truth images. This loss is then backpropagated to update the MLP network’s parameters. The training loss function is given by

(3) $$L=\sum_{r\in ℜ}{\left\|\hat{C}\left(r\right)-C_{gt}\left(r\right)\right\|}_{2}^{2}$$

where ℜ denotes the set of sampled camera rays used for training, $\hat{C}\left(r\right)$ is the rendered color of ray r, and $C_{gt}$ is the corresponding ground-truth pixel color obtained from the captured image. The loss is minimized by updating the parameters of the neural radiance field through backpropagation.

### 2.2. NeRF-Based 3D Reconstruction Scheme for Large-Diameter Rescue Shafts

**Figure 2:**
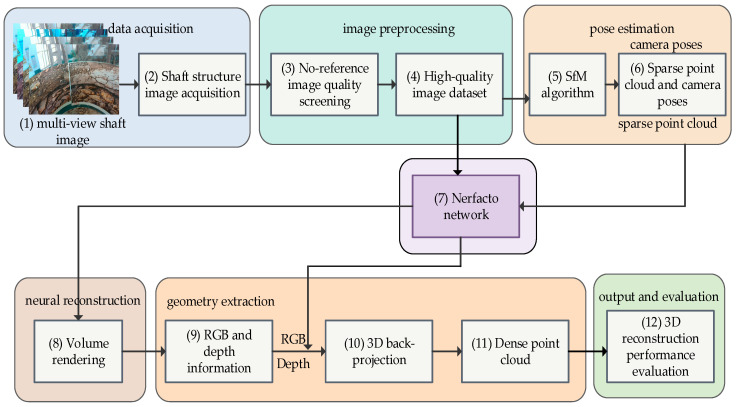
NeRF-based 3D reconstruction scheme.

Figure 2 illustrates the NeRF-based 3D reconstruction scheme for large-diameter rescue shafts. A multi-view dataset covering the entire spatial extent of the shaft is captured using a multi-camera depth imaging system. Subsequently, a high-quality training set is derived through an NR-SNR image filtering strategy. Next, structure-from-motion (SfM) is employed to extract and match features, perform incremental pose estimation, and yield globally consistent camera extrinsics and a sparse 3D point cloud. These outputs are then fed into the Nerfacto network for dense 3D reconstruction. Finally, the geometric accuracy and visual quality of the reconstructed shaft are rigorously evaluated using point cloud analysis and visual inspection.

### 2.3. NR-SNR Image Quality Filtering Strategy

It has been shown in [30,31] that NeRF is relatively insensitive to dataset scale, where merely increasing the number of training images yields only marginal improvements in reconstruction quality while substantially increasing computational cost. As such, we propose an NR-SNR image quality filtering strategy to perform comprehensive data cleaning and refinement. This approach can effectively eliminate blurry, low-texture, and redundant shaft images from the original dataset, thereby constructing a high-quality image dataset and accelerating model convergence.

Let I denote the original shaft image. By applying a uniform 3×3 convolution kernel for mean filtering, the smoothed signal estimate $\hat{S}$ is obtained. Subsequently, the estimated noise component is computed as $\hat{N}=I-\hat{S}$. The variances of the estimated signal and the estimated noise are denoted by $\sigma_{\hat{S}}^{2}$ and $\sigma_{\hat{N}}^{2}$, respectively. Finally, the SNR of the shaft image can be estimated as

(4) $$\mathrm{SNR}=10\times \log_{10}\left(\frac{\sigma_{\hat{S}}^{2}}{\sigma_{\hat{N}}^{2}}\right)$$

**Figure 3:**
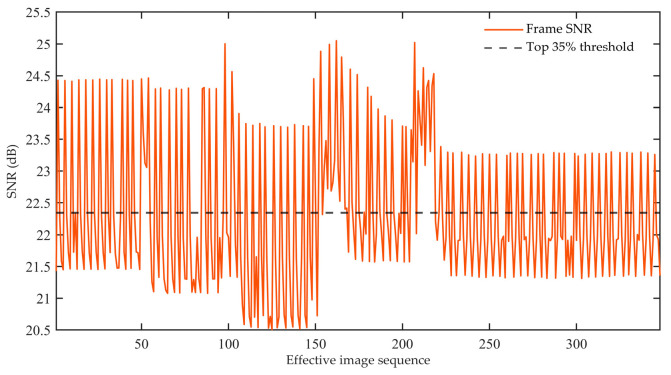
SNR values of individual image frames.

In Figure 3, the evaluated SNR values of each frame in the complete shaft-structure dataset are presented. It intuitively reflects the image-quality characteristics of video frames corresponding to different shaft structures.

All extracted image frames are sorted in descending order by SNR, and the top 35% are retained to construct a high-quality image dataset.

### 2.4. Camera Pose Recovery

After acquiring high-quality wellbore images, we employ an incremental SfM approach for sparse reconstruction to recover a globally consistent camera pose. This effectively avoids solver crashes caused by weak textures and provides drift-free, precise camera parameters and reliable sparse geometric priors for subsequent NeRF implicit field training.

### 2.5. Dense Point Cloud

Conventional NeRFs achieve high-fidelity reconstruction across continuous viewpoints by combining deep neural networks with volumetric rendering. However, they require a large number of training iterations and suffer from slow rendering speeds, which limit their application in real-time engineering. To accelerate 3D reconstruction, we adopt the Nerfacto framework [19,20], a highly optimized variant of NeRF. Due to its capability for minute-level training and real-time rendering, Nerfacto significantly enhances the practicality of NeRF-based shaft reconstruction.

**Figure 4:**
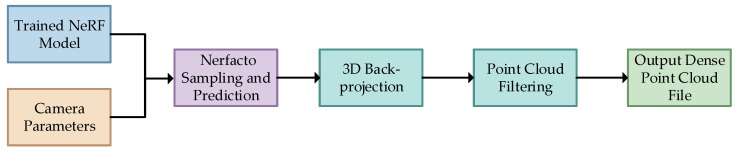
Workflow for dense point-cloud generation.

To assess shaft deformation, the proposed scheme outputs a dense point cloud, as illustrated in Figure 4.

The Nerfacto field predicts and outputs both color information $\left(R,G,B\right)$ and volume density. At the same time, volumetric rendering is employed to simultaneously estimate the expected depth along each ray, thereby generating a high-resolution depth map $D\left(u,v\right)$. Subsequently, 3D back-projection is performed according to (5) to obtain the initial point cloud in the world coordinate system $P_{world}$.

(5) $$P_{world}=R^{-1}\left(K^{-1}D\left(u,v\right)\left[\begin{array}{l} u\\ v\\ 1 \end{array}\right]-t\right)$$

where $P_{world}$ represents the reconstructed three-dimensional point in the world coordinate system; K is the camera intrinsic matrix; $D\left(u,v\right)$ denotes the depth value corresponding to pixel coordinate $\left(u,v\right)$; ${\left[u,v,1\right]}^{T}$ is the homogeneous form of the pixel coordinate; R and t are the rotation matrix and translation vector of the camera extrinsic parameters, respectively.

Meanwhile, Statistical Outlier Removal (SOR) was applied to the preliminary point-cloud model, effectively eliminating floating noise (floaters) and non-surface artifacts introduced during rendering. After that, a surface reconstruction algorithm was employed to generate a high-integrity, dense point cloud of the shaft, preserving accurate spatial coordinates and detailed texture information.

### 2.6. Performance Evaluation Metrics

In this section, we present a comprehensive evaluation of the proposed scheme, encompassing both the accuracy of circle radius fitting and the visual quality metrics of the reconstructed shaft.

#### 2.6.1. Circular Radius Fitting

**Figure 5:**
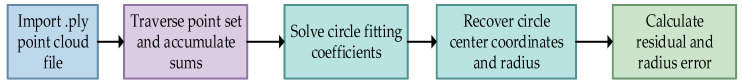
Workflow for circle fitting and radius-error evaluation of the shaft cross-section.

It is well known that the large-diameter rescue shaft is generally cylindrical in shape after 3D reconstruction. To effectively extract its local deformation characteristics, we employ the simplified least-squares circle fitting (Kasa) method to establish a reference circle that quantifies the degree of deformation [32,33,34]. By analyzing the distribution of radial residuals, we subsequently assess the suitability of the shaft as a rescue passage. The simplified Kasa method for the shaft’s dense point cloud is illustrated in Figure 5.

In this study, the manually measured diameter of the simulated rescue shaft was used as the engineering reference dimension for geometric evaluation. The measured diameter was 604 mm, corresponding to a reference radius of 302 mm. This value was used to calculate the relative radius error of the reconstructed cross-sections. It should be noted that this manually measured dimension is not a point-wise ground-truth geometry, such as CAD data or laser-scanned reference data.

The simplified Kasa method of circular radius fitting for the shaft can be described briefly as follows:

Step 1: Import the dense point cloud file. Load the dense point cloud file in .ply format and extract the full set of point coordinates $\left(x,y\right)$.

Step 2: Traverse the point set and perform cumulative summation. Iterate through each point cloud coordinate $\left(x_{i},y_{i}\right)$, and compute the intermediate variables C, D, E, G, and H, which can be expressed as

(6) $$\left\{\begin{array}{l} C=N\sum_{i=1}^{N}x_{i}^{2}-\sum_{i=1}^{N}x_{i}\sum_{i=1}^{N}x_{i}\\ D=N\sum_{i=1}^{N}x_{i}y_{i}-\sum_{i=1}^{N}x_{i}\sum_{i=1}^{N}y_{i}\\ E=N\sum_{i=1}^{N}x_{i}^{3}+N\sum_{i=1}^{N}x_{i}y_{i}^{2}-\sum_{i=1}^{N}\left(x_{i}^{2}+y_{i}^{2}\right)\sum_{i=1}^{N}x_{i}\\ G=N\sum_{i=1}^{N}y_{i}^{2}-\sum_{i=1}^{N}x_{i}\sum_{i=1}^{N}y_{i}\\ H=N\sum_{i=1}^{N}x_{i}^{2}y_{i}+N\sum_{i=1}^{N}y_{i}^{3}-\sum_{i=1}^{N}\left(x_{i}^{2}+y_{i}^{2}\right)\sum_{i=1}^{N}x_{i} \end{array}\right.$$

where *N* denotes the total number of points in the selected cross-sectional segment, and ($x_{i}$, $y_{i}$) represents the two-dimensional coordinates of the i-th point after cross-sectional extraction and projection onto the fitting plane. The summations are performed over all points in the selected cross-section. *C*, *D*, *E*, *G*, and *H* are intermediate accumulated terms used to solve the algebraic least-squares circle-fitting equations.

Step 3: Determine the coefficients of the fitting circle. By substituting the intermediate variables from (6) into (7), the parameters a, b, and c of the fitting circle are derived as

(7) $$\left\{\begin{array}{l} a=\frac{H\cdot D-E\cdot G}{C\cdot G-D^{2}}\\ b=\frac{H\cdot C-E\cdot D}{D^{2}-G\cdot C}\\ c=-\frac{\sum_{i=1}^{N}\left(x_{i}^{2}+y_{i}^{2}\right)+a\sum_{i=1}^{N}x_{i}+b\sum_{i=1}^{N}y_{i}}{N} \end{array}\right.$$

where *a*, *b*, and *c* are the algebraic coefficients of the fitted circle equation $x^{2}+y^{2}+ax+by+c=0$. These coefficients are obtained from the intermediate accumulated terms defined in (6) and are subsequently used to recover the circle center and radius.

Step 4: Recover the circle’s center coordinates and radius. By applying (8), we compute the center coordinates $\left(x_{c},y_{c}\right)$ and the radius r of the fitting circle.

(8) $$\left\{\begin{array}{l} x_{c}=-\frac{a}{2}\\ y_{c}=-\frac{b}{2}\\ r=\frac{1}{2}\sqrt{a^{2}+b^{2}-4c} \end{array}\right.$$

where ($x_{c}$, $y_{c}$) denotes the center coordinates of the fitted circle, and r denotes the fitted radius of the selected shaft cross-section. The fitted radius is later compared with the manually measured reference radius to calculate the relative radius error, while the point-to-circle residuals are used to evaluate the circular consistency of the reconstructed cross-section.

Step 5: Calculate the fitting residual and radius error. For each 5 mm cross-sectional segment, the root mean square error (RMSE) is used to describe the deviation between the reconstructed cross-sectional points and the fitted circle. In this study, the RMSE is used to evaluate the circular fitting consistency of the reconstructed cross-section, rather than the absolute point-wise geometric error with respect to CAD or laser-scanned ground-truth geometry.

(9) $$RMSE_{k}=\sqrt{\frac{1}{N_{k}}\sum_{i=1}^{N_{k}}{\left(\sqrt{{\left(x_{i}-x_{c,k}\right)}^{2}+{\left(y_{i}-y_{c,k}\right)}^{2}}-r_{k}\right)}^{2}}$$

In addition to the fitting residual, the relative radius error was calculated by comparing the fitted radius with the reference radius converted from the manually measured shaft diameter:

(10) $$E_{R}=\frac{\left|r-r_{ref}\right|}{r_{ref}}\times 100\%$$

where r is the fitted radius of the selected cross-sectional segment, and $r_{ref}$ = 302 mm is the reference radius converted from the manually measured diameter of 604 mm.

For further deformation-related analysis, we split the reconstructed shaft point cloud into successive cross-sectional slices parallel to the shaft’s axis, each with a fixed thickness of 5 mm. We calculated relative radius error for each usable slice separately, then averaged these sectional error values to acquire the final statistical error.

#### 2.6.2. Visual Quality Evaluation

In this section, PSNR, SSIM, and LPIPS are used to evaluate the image-level rendering quality of the reconstructed shaft structure from three perspectives: pixel-level fidelity, structural similarity, and perceptual realism. These metrics are not used as direct geometric accuracy indicators. The engineering-oriented geometric accuracy of the reconstructed shaft wall is evaluated separately using circular-section fitting, relative radius error, and fitting residuals.

(1) PSNR

The Peak Signal-to-Noise Ratio (PSNR) is a widely used metric for quantifying the error between an original image and its reconstructed counterpart, with higher PSNR values indicating lower distortion. Mathematically, PSNR is defined as:

(11) $$\mathrm{PSNR}=10\cdot \log_{10}\left(\frac{Max_{I}^{2}}{\mathrm{MSE}}\right)$$

where $Max_{I}^{2}$ is the maximum possible pixel value of the image, e.g., 255 for an 8-bit image, and MSE is the mean squared error used to quantify the pixel-level difference between two images of identical dimensions, which can be expressed by

(12) $$\mathrm{MSE}=\frac{1}{MN}\sum_{i=1}^{M}\sum_{j=1}^{N}{\left[I\left(i,j\right)-\hat{I}\left(i,j\right)\right]}^{2}$$

where $I\left(i,j\right)$ and $\hat{I}\left(i,j\right)$ are the pixel values of the original and reconstructed images, respectively, and M and N are the height and width of the image.

(2) SSIM

The structural similarity index (SSIM) is a comprehensive metric for assessing structural similarity, incorporating perceptual attributes of the human visual system across three domains: luminance, contrast, and structural information. Its value ranges from 0 to 1, where a value closer to 1 indicates greater similarity between the two images. The index can be expressed as

(13) $$\mathrm{SSIM}\left(x,y\right)=\frac{\left(2\mu_{x}\mu_{y}+C_{1}\right)\left(2\sigma_{xy}+C_{2}\right)}{\left(\mu_{x}^{2}+\mu_{y}^{2}+C_{1}\right)\left(\sigma_{x}^{2}+\sigma_{y}^{2}+C_{2}\right)}$$

where x and y are the two images to be compared; $\mu_{x}$ and $\mu_{y}$ are the local mean values of images x and y; $\sigma_{x}^{2}$ and $\sigma_{y}^{2}$ are the local variances of images x and y; $\sigma_{xy}^{2}$ is the local covariance between images x and y; $C_{1}$ and $C_{2}$ are constants introduced to avoid division by zero, typically defined as $C_{1}={\left(K_{1}L\right)}^{2}$ and $C_{2}={\left(K_{2}L\right)}^{2}$, where L represents the dynamic range of pixel values, $K_{1}=0.01$ and $K_{2}=0.03$ are empirical constants.

(3) LPIPS

We adopt learned perceptual image patch similarity (LPIPS) as a perceptual-level metric for evaluating the rendering quality of the 3D reconstruction of the shaft structure, defined as (14). Its value ranges from 0 to 1, with lower values indicating greater perceptual similarity between the two images.

(14) $$\mathrm{LPIPS}\left(I_{1},I_{2}\right)=\sum_{l}w_{l}\frac{1}{H_{l}W_{l}}\sum_{h,w}{\left\|\phi_{l}{\left(I_{1}\right)}_{h,w}-\phi_{l}{\left(I_{2}\right)}_{h,w}\right\|}_{2}^{2}$$

where $\phi_{l}\left(\cdot \right)$ is the feature extraction function of the l layer; $H_{l}$ and $W_{l}$ are the height and width of the feature map at that layer, respectively; $w_{l}$ represents the weights learned from large-scale perceptual experiments.

### 2.7. Implementation Details

To provide a clearer description of the experimental configuration, the main implementation details of the proposed NeRF scheme are summarized in this section. The proposed NeRF pipeline was implemented using the Nerfstudio framework, with Nerfacto adopted as the backbone model. Unless otherwise specified, the main reconstruction experiments were trained for 100,000 iterations. The main training settings and optimization parameters are listed in Table 1.

As shown in Table 1, the proposed NeRF scheme was trained for 100,000 iterations using the Nerfacto model. A 90%/10% train/evaluation split was adopted, and 4096 rays were sampled per batch for both training and evaluation. Mixed-precision training and normal prediction were enabled in the tested configuration. The trained model was then exported as a dense point cloud with 4,000,000 points for subsequent filtering, cross-sectional extraction, and circular-section fitting.

## 3. Results

In this section, we evaluate the performance of the proposed NeRF-based scheme on a laboratory-constructed large-diameter rescue shaft simulation platform, using the MVS and 3DGS schemes as benchmarks.

### 3.1. Experimental Environment

**Figure 6:**
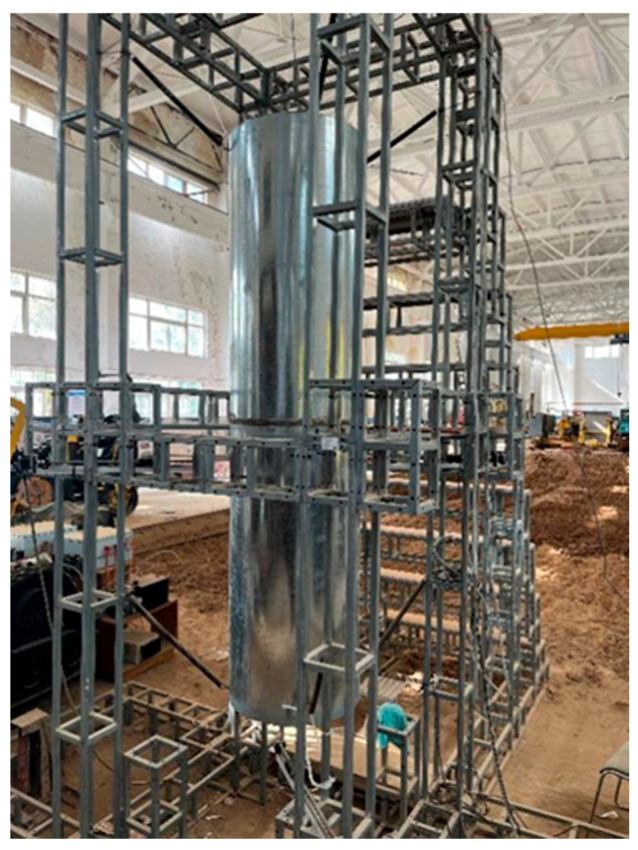
Experimental platform for the large-diameter rescue shaft.

As shown in Figure 6, the experimental platform consists of four primary components: a top guide wheel, a simulated rescue shaft, a hoisting unit, and a simulated rescue capsule. The shaft measures 2500 mm in depth and 604 mm in diameter. Four D435i depth cameras are installed at the bottom center of the rescue capsule to capture real-time images of the shaft interior during both upward and downward motions. Detailed specifications of the experimental platform are provided in Table 2.

The image acquisition protocol followed the multi-camera imaging setup used in the experimental platform described above. Four Intel RealSense D435i cameras were used to acquire multi-view RGB images of the shaft inner wall, with an image resolution of 1280 × 720 pixels and a frame rate of 30 FPS. During acquisition, the camera platform moved along the shaft axis, and the image streams from different cameras were stored separately with timestamp information for subsequent alignment and SfM pose estimation.

The original dataset contained 348 images. After NR-SNR image quality filtering, 123 retained images were used as the main image set for the proposed NeRF reconstruction pipeline. The manually measured shaft diameter was 604 mm, corresponding to a reference radius of 302 mm for geometric evaluation.

In the shaft experiment, the camera intrinsic matrix and the estimated extrinsic matrix are defined as follows, respectively.

(15) $$K_{1}=\left[\begin{array}{ccc} 924.896 & 0 & 652.304\\ 0 & 924.055 & 359.444\\ 0 & 0 & 1 \end{array}\right]$$

(16) $$\left[\left.R_{1}\right|\xi_{1}\right]=\left[\begin{array}{cccc} -0.986 & \;\;\;0.107 & -0.124 & 0.081\\ -0.162 & -0.754 & \;\;\;0.636 & 2.597\\ -0.025 & \;\;\;0.648 & \;\;\;0.761 & 2.492 \end{array}\right]$$

where $K_{1}$ denotes the intrinsic matrix of the first camera, with the diagonal elements representing the focal lengths and the last column containing the principal-point coordinates. $R_{1}$ and $\xi_{1}$ denote the estimated rotation matrix and translation vector of the first camera, respectively.

### 3.2. Ablation Study of NR-SNR Image Filtering

#### 3.2.1. Effect of NR-SNR Retention Ratio on SfM

To validate the impact of the NR-SNR image quality filtering strategy on the efficiency and geometric consistency of SfM sparse reconstruction, we conducted a retention-ratio ablation experiment. Specifically, SfM sparse reconstruction was performed under identical hardware conditions and SfM parameter settings using four image-retention ratios, i.e., 15%, 35%, 55%, and 100%. Among them, the 100% setting represents the unfiltered full dataset with 348 original images, whereas the 35% setting represents the NR-SNR-filtered high-quality dataset with 123 retained images. This setting ensures a fair comparison of SfM performance under different image-retention levels. The results are presented in Table 3.

As shown in Table 3, increasing the NR-SNR image retention ratio generally increases the number of sparse points, but the computational cost grows much faster than the sparse geometric gain. When only 15% of the images were retained, SfM reconstruction was completed within 0.629 min, but only 13,150 sparse points were generated, indicating insufficient sparse geometric coverage. When the retention ratio increased to 35%, 123 images were registered and 20,237 sparse points were reconstructed within 4.521 min, showing a substantial improvement in sparse geometric coverage while maintaining a low computational cost.

Further increasing the retention ratio to 55% increased the number of sparse points only moderately to 23,206, whereas the reconstruction time increased to 23.349 min. When all images were used, the reconstruction time reached 197.044 min, approximately 43.58 times that of the 35% setting, while the number of sparse points increased only by 1.44 times. In addition, more frequent CHOLMOD/Eigen warnings were observed in the high-retention settings, indicating a heavier numerical burden during bundle adjustment. This suggests that the additional points generated by excessive redundant images do not provide proportional geometric benefits but instead increase the computational burden of SfM optimization.

Based on these results, the 35% retention ratio was selected as a practical trade-off between SfM reconstruction efficiency, sparse geometric coverage, and numerical stability for the present rescue-shaft dataset. It should be noted that this value is not treated as a universal optimal threshold. Instead, it is an empirically validated setting under the high-overlap, weak-texture, and locally blurred imaging conditions of the simulated rescue-shaft dataset. The filtered image set preserves sufficient global geometric priors for subsequent Nerfacto training while significantly reducing redundant and low-quality constraints in SfM pose estimation.

#### 3.2.2. Effect of NR-SNR Retention Ratio on Neural Rendering Quality

To further validate the impact of the NR-SNR image retention ratio on downstream NeRF reconstruction quality, we conducted a rendering-quality evaluation experiment. Specifically, Nerfacto models were trained using image subsets generated with four retention ratios (15%, 35%, 55%, and 100%) under identical training settings. The rendered images were compared with the corresponding reference images using peak signal-to-noise ratio (PSNR), structural similarity index (SSIM), and learned perceptual image patch similarity (LPIPS). The results are presented in Table 4.

As shown in Table 4, the 15% setting caused a clear drop across all three rendering metrics, indicating that overly aggressive image reduction undermines the view coverage essential for stable Nerfacto training. The 55% setting yielded the highest PSNR and a marginally better SSIM than the 35% setting. However, this PSNR improvement and marginal SSIM gain came at a disproportionate computational cost, with the SfM time increasing from 4.521 min to 23.349 min, and did not translate into a perceptual improvement, as indicated by the worse LPIPS score. Notably, the 35% setting achieved the lowest LPIPS among all tested ratios, suggesting better perceptual consistency despite a slightly lower PSNR.

The 100% setting did not further improve the rendering quality despite using all input images, suggesting that simply increasing the number of images does not necessarily enhance Nerfacto reconstruction quality after sufficient view coverage has been achieved. Therefore, considering both the SfM results in Table 3 and the rendering-quality results in Table 4, the 35% retention ratio was selected as a practical efficiency–quality trade-off for the present rescue-shaft dataset, rather than as a universal optimal threshold.

### 3.3. Geometric Accuracy Analysis

Unlike the rendering-quality metrics reported in Section 3.4, the geometric evaluation in this section focuses on the physical dimensional consistency of the reconstructed shaft wall. Specifically, the fitted radius was compared with the manually measured reference radius, while the fitting residual was used to describe the circular consistency of each reconstructed cross-section.

#### 3.3.1. Shaft Radius Fitting Accuracy

In this study, we investigate the performance of the proposed simplified Kasa method. In particular, we select cross-sectional point-cloud data from the 600–605 mm depth interval of the simulated large-diameter rescue shaft for evaluation.

**Figure 7:**
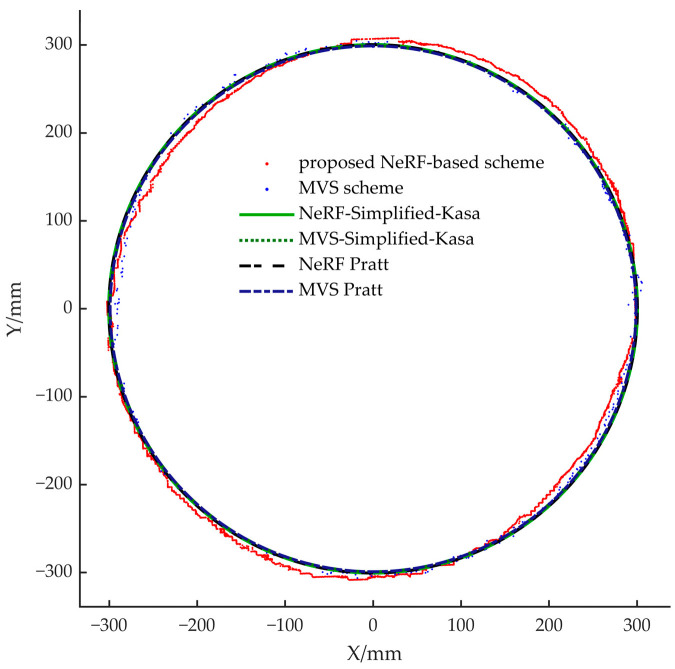
Radius fitting results for the rescue shaft cross-section within the 600–605 mm depth interval.

Figure 7 compares the shaft radius-fitting accuracy of the proposed NeRF-based 3D reconstruction scheme with that of the conventional MVS-based scheme. Both schemes are evaluated using the simplified Kasa method, and the Pratt method is additionally included as a reference. The curves labeled NeRF Simplified Kasa and MVS Simplified Kasa represent the fitting results obtained by the proposed NeRF-based and conventional MVS-based schemes, respectively, when the simplified Kasa method is used. The curves labeled NeRF Pratt and MVS Pratt show the corresponding results obtained using the Pratt method.

Figure 7 shows that both the simplified Kasa and Pratt methods accurately fit the shaft’s circular radius, with high congruence. It is also shown in Figure 7 that the extracted cross-sectional point clouds of the shaft display high completeness and closure, without apparent geometric defects, thereby ensuring the reliability of the fitted radius.

To further quantify reconstruction accuracy, the circle-radius fitting errors of the proposed NeRF-based scheme, along with comparisons with the conventional MVS scheme at cross-sections corresponding to different shaft heights, are listed in Table 5. The conventional MVS scheme is evaluated based on a single reconstruction. In contrast, because implicit radiance field methods inherently involve stochasticity during network initialization and ray sampling, we mitigate the effects of randomness in the proposed NeRF-based scheme by averaging results from multiple independent reconstructions.

The proposed NeRF-based scheme outperforms the conventional MVS scheme. From a practical viewpoint, the proposed NeRF-based scheme with the simplified Kasa method reduces the fitting error from 1.466% to 0.878%, achieving an improvement of around 40.1% over the conventional MVS scheme. Additionally, the proposed NeRF-based scheme with the Pratt method yields about 36.6% performance improvement over the conventional MVS scheme. This improvement can be attributed to the implicit radiance field model’s ability to learn high-dimensional feature representations that more accurately capture the geometric characteristics of cylindrical structures. In particular, the proposed NeRF-based scheme outperforms the feature-matching-based MVS scheme in recovering fine details within weak-texture regions.

Furthermore, based on the analysis above, we can see that the simplified Kasa method achieves the same performance as the Pratt method. However, due to its high computational efficiency and simple implementation, the simplified Kasa method is more suitable for practical engineering applications. More explicitly, the simplified Kasa method shows almost no performance degradation while imposing significantly lower computational complexity than the Pratt benchmark. Thus, without loss of generality, the simplified Kasa method is consistently employed for quantitative assessment in the subsequent validation of the NeRF-based scheme for monitoring real structural deformations of the shaft.

#### 3.3.2. Shaft Deformation Analysis

**Figure 8:**
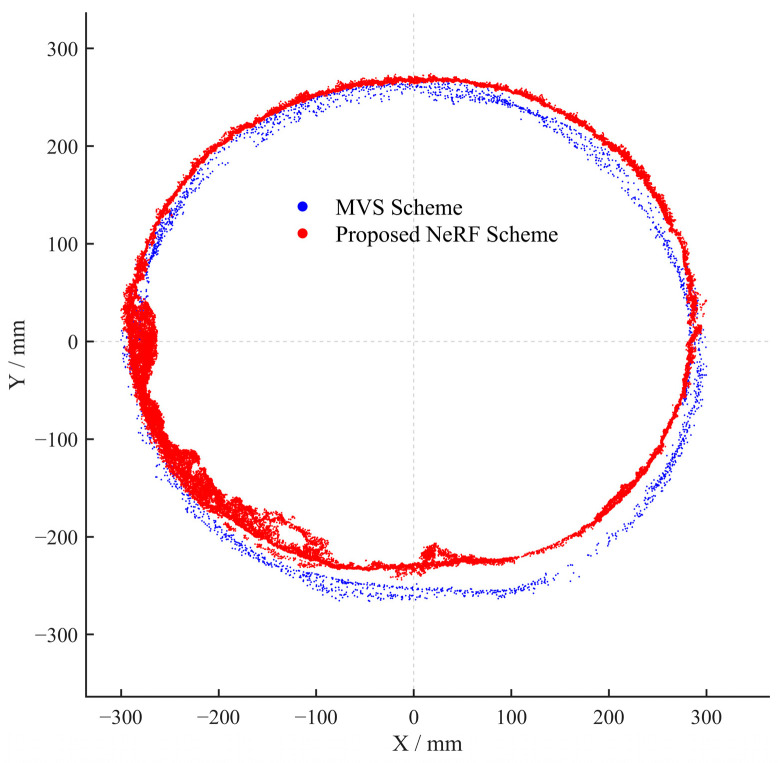
Deformation trend of the rescue shaft. Note: The results of the conventional MVS scheme are plotted as blue points, whereas those of the proposed NeRF scheme are shown as red points.

**Figure 9:**
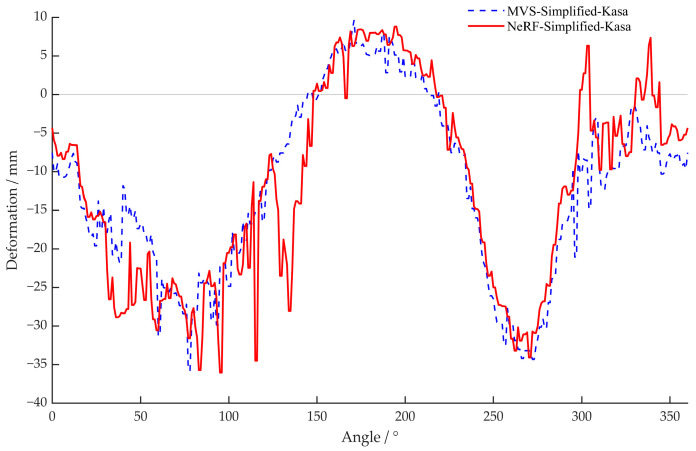
Radial deformation of the rescue shaft.

We now look at the effectiveness of the proposed NeRF-based scheme for monitoring structural deformation in rescue shafts. For this purpose, we designed a dedicated apparatus specifically for simulating deformation. The apparatus features a lead screw circumferentially arranged along the shaft’s outer wall, driven by a stepper motor to achieve a linear displacement of 35 mm. The deformed cross-sectional point clouds are captured using a depth camera for subsequent 3D reconstruction. Figure 8 shows the results of the circle fitting applied to the deformed rescue shaft. In contrast, Figure 9 illustrates the difference between the point-cloud radius and the original shaft radius, defined as the deformation magnitude. From a physical perspective, a negative value indicates compressive deformation of the rescue shaft, whereas a positive value signifies expansive deformation.

It can be seen from Figure 8 and Figure 9 that the progressive exertion of thrust from the stepper motor induces a pronounced non-circular compressive deformation in the rescue shaft, causing the cross-section to deviate from a regular circular shape. Both the conventional MVS and NeRF-based schemes accurately capture and quantify this deformation. Moreover, the deformation curves derived from both approaches exhibit a fundamentally consistent trend. For example, the conventional MVS scheme identifies a maximum compressive displacement of 35 mm at an azimuth of approximately 270°, and a maximum expansive displacement of 9 mm at an azimuth of approximately 175°. These measurements yield a maximum clearance diameter of 534 mm. In comparison, the proposed NeRF-based scheme detects a slightly higher maximum compressive displacement of 36 mm, while the maximum expansive displacement remained consistent at 9 mm, resulting in a maximum clearance diameter of 532 mm. It should also be stressed that the discrepancy between the two methods is minimal, with a 1 mm difference in the maximum compression measurement and a 2 mm difference in the maximum clearance diameter, corresponding to a relative error of less than 0.4%. Thus, these results imply that the proposed NeRF-based scheme is feasible and highly accurate for deformation detection in large-diameter rescue shafts.

### 3.4. Visual Reconstruction Quality

**Figure 10:**
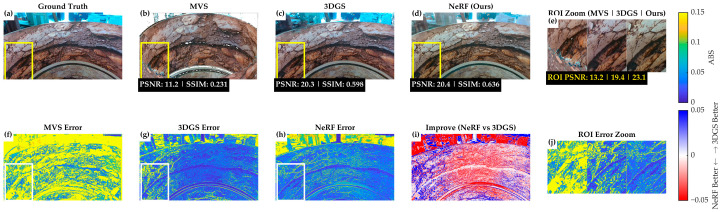
Comparison of 3D reconstruction results in high-texture regions. (**a**) Ground-truth image; (**b**) conventional MVS result; (**c**) 3DGS result; (**d**) proposed NeRF-based result; (**e**) enlarged ROI comparison among MVS, 3DGS, and the proposed method; (**f**–**h**) absolute error maps of MVS, 3DGS, and the proposed method, respectively; (**i**) improvement map comparing the proposed method with 3DGS; (**j**) enlarged ROI error comparison. The yellow boxes indicate the selected ROI regions in the reconstructed images, the white boxes indicate the corresponding ROI regions in the error maps, and the arrow in the color bar indicates the direction of relative improvement between the proposed NeRF-based method and 3DGS.

**Figure 11:**
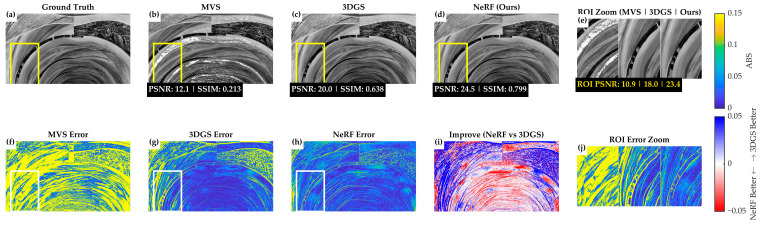
Comparison of 3D reconstruction results in low-texture regions. (**a**) Ground-truth image; (**b**) conventional MVS result; (**c**) 3DGS result; (**d**) proposed NeRF-based result; (**e**) enlarged ROI comparison among MVS, 3DGS, and the proposed method; (**f**–**h**) absolute error maps of MVS, 3DGS, and the proposed method, respectively; (**i**) improvement map comparing the proposed method with 3DGS; (**j**) enlarged ROI error comparison. The yellow boxes indicate the selected ROI regions in the reconstructed images, the white boxes indicate the corresponding ROI regions in the error maps, and the arrow in the color bar indicates the direction of relative improvement between the proposed NeRF-based method and 3DGS.

To assess the 3D reconstruction quality of different methods under varying texture conditions, Figure 10 and Figure 11 present visual comparisons in representative high-texture and low-texture regions of the rescue shaft. In addition to the reconstructed images, local ROI enlargements and error heatmaps are also provided to illustrate differences in detail recovery, structural continuity, and spatial error distribution among the conventional MVS scheme, the 3DGS baseline, and the proposed NeRF-based scheme.

Figure 10 and Figure 11 show that all three methods can recover the main geometric outline of the shaft in high-texture regions. At the same time, noticeable differences remain in local detail fidelity and error distribution. The conventional MVS scheme shows more local discontinuities and incomplete details than the proposed NeRF-based scheme. The proposed NeRF-based scheme produces visually more complete and coherent reconstruction results, with clearer surface structures and fewer locally degraded regions. This improvement can be attributed to the implicit photometric consistency constraints of the radiance-field representation, which help to interpolate weak-texture regions more smoothly. This advantage becomes more evident in low-texture areas, where large homogeneous surfaces and weak feature variations make reconstruction more challenging. Meanwhile, the 3DGS scheme also exhibits locally unstable reconstruction in some boundary and smooth-surface regions. By contrast, NeRF maintains better visual continuity and better preserves the cylindrical contour and surface appearance of the shaft wall.

To further observe the local reconstruction errors of different schemes, magnified regions of interest (ROIs) and the corresponding error heatmaps are shown in Figure 10 and Figure 11. In the heatmaps, warmer colors represent larger errors, while cooler colors represent smaller errors. It is shown in Figure 10 and Figure 11 that the conventional MVS and 3DGS schemes produce more concentrated error regions in homogeneous shaft-wall areas, structural transition regions, and local detail regions. This indicates that these two schemes are more easily affected by local distortion and discontinuity when the shaft-wall texture is weak. In contrast, the proposed NeRF-based scheme shows fewer high-error regions and a smoother error distribution. This result suggests that the proposed scheme can better preserve the continuity of the shaft-wall surface and local details under the low-texture and low-illumination rescue-shaft condition.

Following the qualitative analysis, we further performed a quantitative evaluation of image-level rendering quality using PSNR, SSIM, and LPIPS. To clarify the evaluation protocol and avoid relying on a single best-performing result, the visual-quality metrics of the 3DGS and proposed NeRF-based schemes were reorganized using repeated-trial statistics. In each trial, the metric value was first averaged over the same test image set, and the final results are reported as mean ± standard deviation over three repeated trials. Since the conventional MVS scheme was evaluated based on a single fixed reconstruction under the same parameter settings, its visual metrics are reported as single values. The results are listed in Table 6.

As shown in Table 6, the proposed NeRF-based scheme achieves higher image-level quality than the conventional MVS scheme in both high-texture and low-texture scenarios. In the low-texture scenario, the proposed NeRF-based scheme improves PSNR by about 13.03 dB, increases SSIM by approximately 0.6497, and reduces LPIPS by about 0.6885 compared with the conventional MVS scheme. This indicates that the radiance-field-based reconstruction is more suitable than conventional MVS for preserving visual consistency in weak-texture shaft-wall regions.

Compared with the 3DGS baseline under the unified repeated-trial evaluation protocol, the proposed NeRF-based scheme also shows better overall visual-quality performance. In the high-texture scenario, the proposed NeRF-based scheme achieves a PSNR improvement of about 1.29 dB and an SSIM increase of approximately 0.1077, while the LPIPS values of the two neural rendering schemes are very close. In the low-texture scenario, the proposed NeRF-based scheme obtains a PSNR of 23.67 dB and an SSIM of 0.7766, which are about 3.77 dB and 0.1346 higher than those of 3DGS, respectively. Meanwhile, the LPIPS value is reduced from 0.1891 to 0.1700, indicating better perceptual consistency under weak-texture conditions.

It should be noted that the standard deviations in Table 6 describe the variation in the visual-quality metrics across three repeated trials. Therefore, these values are used as descriptive statistics for repeated-trial evaluation, rather than as formal statistical significance analysis. Overall, the results show that the proposed NeRF-based scheme maintains favorable visual-quality performance under the tested rescue-shaft conditions. In addition, PSNR, SSIM, and LPIPS only evaluate image-level rendering quality, while the geometric accuracy and deformation-measurement applicability of the reconstructed shaft wall are evaluated separately in Section 3.3 using relative radius error and circular fitting residuals.

### 3.5. Comprehensive Analysis of Efficiency and Reliability

While geometric accuracy and visual quality are fundamental evaluation metrics, reconstruction efficiency is also a pivotal factor in determining the engineering applicability of 3D reconstruction schemes. In particular, for rescue-shaft inspection, the reconstruction result is expected not only to be generated within an acceptable time cost but also to provide a geometrically usable output for subsequent deformation measurement. Therefore, in this section, the computational performance and output applicability of the main reconstruction stages and comparison methods are further summarized. The runtime, hardware platform, memory consumption, and output scale are listed in Table 7.

As shown in Table 7, the NR-SNR image filtering strategy substantially reduced the computational burden of SfM sparse reconstruction. When all 348 images were used, SfM reconstruction required 197.044 min and generated 29,210 sparse points. In contrast, the 35% retention setting reduced the SfM runtime to 4.521 min while still generating 20,237 sparse points. This indicates that removing redundant and low-quality images can improve the efficiency of SfM pose estimation without a proportional reduction in sparse geometric coverage.

For neural-field reconstruction, the 3DGS baseline required 31 min on the RTX 4070 GPU, showing an advantage in absolute runtime owing to its explicit Gaussian representation and rasterization-based rendering mechanism. The proposed NeRF scheme required 65 min on the same GPU, with an approximate peak GPU memory consumption of 9 GB and system memory consumption of 22 GB. From the perspective of runtime alone, 3DGS is more efficient. However, as discussed in Section 3.4, 3DGS is more dependent on the quality of the initial point cloud, which may affect the structural completeness of the reconstructed result in weak-texture and low-illumination shaft-wall scenes.

In contrast, the proposed NeRF scheme produced a dense point-cloud output with 4,000,000 points, which could be further used for point-cloud filtering, cross-sectional extraction, and circular-section fitting. Although it required a longer runtime than the 3DGS baseline, its output was more directly compatible with the subsequent radius-fitting-based deformation assessment used in this study.

The representative NeuS baseline further illustrates that computational cost alone cannot fully determine the practical applicability of a reconstruction method. Under the tested configuration, NeuS required 169.67 min on an NVIDIA A100 GPU. Although its final-stage loss remained relatively stable, radius fitting was not performed because the reconstructed output did not provide a shaft-wall geometry suitable for direct circular-section measurement, as discussed in Section 3.6.

Therefore, considering SfM efficiency, neural-field reconstruction cost, memory consumption, output scale, and measurement applicability, the proposed NeRF scheme provides a practical balance for the present rescue-shaft dataset. Compared with the faster 3DGS baseline, it requires additional reconstruction time but provides a point-cloud output that is more suitable for engineering-oriented geometric measurement. Compared with the tested NeuS baseline, it required less runtime under the tested configuration and provided an output more compatible with the subsequent circular-section fitting workflow.

### 3.6. Evaluation of the NeuS Baseline

To further evaluate the applicability of modern implicit surface reconstruction methods to rescue-shaft inner-wall reconstruction, NeuS was additionally tested as a representative SDF-based baseline. In particular, the NeuS model was trained using the same rescue-shaft image data under the tested baseline configuration. The purpose of this comparison was not to exhaustively optimize all SDF-based variants, but to examine whether a standard SDF-based implicit surface formulation can be directly applied to the hollow cylindrical inner-wall reconstruction task required for subsequent geometric measurement.

Different from the proposed NeRF scheme, which outputs a dense point cloud for circular-section fitting, the NeuS baseline produced a surface representation that did not adequately preserve the shaft-wall geometry under the tested configuration. Therefore, direct radius-fitting accuracy was not further compared for the NeuS result. Instead, this section compares the two neural reconstruction methods in terms of training configuration, computational cost, convergence behavior, and measurement applicability. The results are summarized in Table 8.

As shown in Table 8, the proposed NeRF scheme completed 100,000 training iterations on an RTX 4070 GPU within 65 min and produced a point-cloud output that could be further used for circular-section fitting and deformation assessment. In contrast, the NeuS baseline required 100,000 training iterations on an NVIDIA A100 GPU, with a longer runtime of 169.67 min. Although the final-stage loss of NeuS remained relatively stable, its output was not suitable for direct circular-section measurement of the rescue-shaft wall.

This result can be attributed to the different geometric formulations of the two methods. NeuS reconstructs geometry by learning a continuous signed-distance field and extracting the zero-level surface, which is generally effective for object-centric scenes with clear surface boundaries and sufficient multi-view constraints. However, the rescue-shaft scene in this study is an elongated hollow cylindrical inner-wall structure with weak and repetitive textures. Under such conditions, the inside–outside relationship, scale normalization, and zero-level surface constraint become less straightforward, making direct SDF-based surface recovery more challenging under the tested baseline configuration. Meanwhile, the NeuS baseline required more training iterations and a longer runtime, but its output was still not suitable for subsequent radius-fitting-based deformation analysis. Therefore, considering both computational efficiency and measurement applicability, the proposed NeRF scheme was adopted as the main reconstruction framework because it provides a more practical point-cloud output for engineering-oriented geometric measurement.

## 4. Discussion

The present investigation reveals a notable characteristic of rescue-shaft image acquisition: the captured image sequence usually contains high image overlap, weak texture, and constrained illumination. The experimental results indicate that increasing the number of input images does not necessarily lead to continuous improvement in reconstruction quality. Instead, blindly increasing the input scale may introduce redundant observations and increase the computational burden of SfM pose estimation. In the simulated rescue-shaft dataset, the NR-SNR image quality filtering strategy retained approximately 35% high-quality images, which preserved the effective observations required for global cylindrical-geometry recovery while reducing the influence of redundant or low-quality frames. Therefore, this filtering strategy provides a practical efficiency–quality trade-off for the present dataset and supports the subsequent NeRF-based reconstruction process.

The circular fitting results further indicate that the proposed NeRF-based scheme reduces the mean fitting error compared with the conventional MVS scheme. In low-texture regions, the conventional MVS scheme is more prone to sparse point-cloud distribution, local noise, and discontinuous reconstruction, mainly because it relies strongly on explicit feature extraction and matching. In comparison, the proposed NeRF-based scheme provides a more continuous representation of the shaft-wall surface in weak-texture regions, which is beneficial for circular-section fitting and deformation-related measurement. These results suggest that the proposed scheme is suitable for static shaft-wall reconstruction and can provide useful geometric information for deformation assessment and passability evaluation.

Further comparison shows that 3DGS has an advantage in reconstruction speed, whereas the proposed NeRF-based scheme provides a dense point-cloud output that is more directly compatible with the subsequent circular-section fitting workflow. The 3DGS baseline is more dependent on the quality of the initial sparse point cloud, which may affect structural completeness in weak-texture and low-illumination shaft-wall scenes. Although the proposed NeRF-based scheme requires a longer reconstruction time, it provides a more suitable output for engineering-oriented geometric measurement in the tested rescue-shaft dataset. Therefore, reconstruction speed alone cannot fully determine the applicability of a method for rescue-shaft deformation assessment; model completeness, geometric usability, and compatibility with subsequent measurement procedures should also be considered.

It should also be noted that the circular-section fitting used in this study mainly evaluates the overall dimensional consistency of the reconstructed shaft cross-sections. In practical rescue operations, trapped personnel are not lifted directly through the shaft wall; instead, they are transported by a rescue capsule with an approximately circular cross-section. Therefore, circular-section fitting is used in this study not to assume that all local deformations are perfectly circular, but to provide an engineering-oriented indicator of whether the reconstructed shaft cross-section can support rescue-capsule passage. This assumption is appropriate for rescue shafts that approximately preserve a cylindrical structure, and it allows the relative radius error and fitting residuals to be quantified in an intuitive manner. However, a single circular fitting model may not fully describe highly irregular local deformation, such as local inward protrusions, non-uniform dents, or strongly non-circular cross-sectional changes. For such cases, circular fitting should be combined with local radial-deviation analysis and maximum passable-radius estimation.

This limitation does not mean that the reconstructed point cloud cannot be used for non-standard deformation analysis. Previous rescue-shaft studies have shown that local protrusions and non-standard deformation can be identified by analyzing the point-cloud radius distribution and the maximum inscribed circle of each cross-section [34]. Therefore, in future work, the proposed reconstruction framework can be further combined with local radial-profile analysis, maximum passable-radius estimation, and surface-based deformation metrics to describe more complex shaft-wall deformation.

The present validation was mainly conducted on a physical rescue-shaft simulation platform. This platform can reproduce several typical characteristics of shaft interiors, including confined space, weak surface texture, and limited illumination, and thus provides a controllable basis for evaluating different 3D reconstruction schemes. However, real mine environments may involve additional disturbances such as dust, water mist, object occlusion, device vibration, and more complex illumination variations. In addition, NeuS was evaluated only as a representative SDF-based baseline under the tested configuration, and this comparison does not exhaustively cover all SDF-based or neural surface reconstruction variants. Therefore, further validation using real rescue-shaft data and more challenging field conditions is still necessary to assess the generalization capability and practical application boundaries of the proposed scheme.

## 5. Conclusions

In this paper, a NeRF-based 3D reconstruction scheme was developed for large-diameter rescue-shaft inner-wall reconstruction and deformation-oriented geometric measurement. The proposed workflow combines NR-SNR image quality filtering, SfM pose estimation, Nerfacto-based implicit reconstruction, point-cloud export, and circular-section fitting.

The NR-SNR retention-ratio experiment shows that retaining approximately 35% high-quality images provides a practical efficiency–quality trade-off for the present dataset. Compared with using all original images, this setting substantially reduces the SfM computational burden while preserving sufficient sparse geometric information for subsequent reconstruction.

The experimental results on the simulated rescue-shaft platform demonstrate that the proposed NeRF-based scheme provides favorable visual reconstruction quality and geometric measurement applicability under weak-texture and low-illumination conditions. Compared with the conventional MVS scheme and the tested 3DGS baseline, the proposed scheme produces a point-cloud output that is more suitable for subsequent circular-section fitting and radius-error evaluation. In addition, the representative NeuS baseline experiment indicates that direct SDF-based surface recovery remains challenging for the tested hollow cylindrical shaft-wall scene.

Future work will focus on validating the proposed scheme using real rescue-shaft data and more complex field conditions, including dust, water mist, vibration, occlusion, and illumination variations. In addition, local radial-deviation analysis, maximum passable-radius estimation, and more flexible surface-based deformation metrics will be further investigated to improve the evaluation of irregular shaft-wall deformation.

## Figures and Tables

**Table 1:** Implementation Details of the Proposed NeRF Scheme.

Item	Setting	Item	Setting
Framework	Nerfstudio	Backbone model	Nerfacto
Training iterations	100,000	Train/evaluation split	90%/10%
Batch size	4096 rays	Optimizer	Adam
Field learning rate	0.01	Proposal network LR	0.01
Camera optimizer LR	0.001	Final learning rate	0.0001
Mixed precision	Enabled	Normal prediction	Enabled
Normal loss weight	0.01	GPU/runtime	RTX 4070/65 min
Peak GPU memory	approx. 9 GB	System memory	approx. 22 GB
Exported points	4,000,000	Post-processing	Filtering and circular-section fitting

Note: Since Nerfacto is optimized by training iterations rather than conventional epochs, the number of training iterations is reported. The batch size refers to the number of sampled rays per batch. The complete configuration file is provided in the public repository.

**Table 2:** Basic parameters of the experimental platform.

**Item**	**Specification**
Programming language	Python 3.8.20
Development environment	PyCharm 2020.1.3(x64)
Operating system	Windows 10
CPU	Intel Core I7-12700
GPU	NVIDIA GeForce RTX 4070

**Table 3:** Impact of NR-SNR Image Retention Ratio on SfM Sparse Reconstruction.

Retention (%)	Images/Reg.	SfM Time (min)	Sparse Points	Time Ratio	Status
15	52/52	0.629	13,150	0.14×	S1
35	123/123	4.521	20,237	1.00×	S2
55	192/192	23.349	23,206	5.16×	S3
100	348/348	197.044	29,210	43.58×	S4

Notes: Reg. denotes registered images. Time ratio is calculated using the 35% retention setting as the reference. S1: successful reconstruction with limited sparse coverage; S2: successful reconstruction with manageable solver burden; S3: successful reconstruction with increased numerical warnings; S4: frequent CHOLMOD/Eigen warnings and the highest computational burden.

**Table 4:** Impact of NR-SNR Image Retention Ratio on Nerfacto Rendering Quality.

Retention (%)	Test Images	PSNR ↑	SSIM ↑	LPIPS ↓
15	5	24.1312	0.7519	0.2097
35	13	24.6700	0.8506	0.1703
55	19	27.5192	0.8514	0.1844
100	34	26.5505	0.7973	0.1951

Note: For each retention ratio, approximately 10% of the retained image subset was used for testing; therefore, the number of test images varied with the retention ratio. The arrows indicate the preferred direction of each metric: higher values are better for PSNR and SSIM, whereas lower values are better for LPIPS.

**Table 5:** Shaft radius fitting errors for different methods.

	Simplified Kasa (%)	Pratt (%)
Conventional MVS	1.466%	1.476%
NeRF	0.878%	0.936%

**Table 6:** Visual-quality metrics of different reconstruction schemes.

Scheme	Texture Scenario	PSNR/dB ↑	SSIM ↑	LPIPS ↓
MVS	High-texture	11.53	0.1745	0.6874
	Low-texture	10.64	0.1269	0.8585
3DGS	High-texture	21.88 ± 0.1196	0.5948 ± 0.0022	0.3830 ± 0.0162
	Low-texture	19.90 ± 0.0107	0.6420 ± 0.0005	0.1891 ± 0.0002
NeRF-based	High-texture	23.17 ± 0.54	0.7025 ± 0.0305	0.3820 ± 0.0067
	Low-texture	23.67 ± 0.96	0.7766 ± 0.0707	0.1700 ± 0.0786

Note: The conventional MVS scheme was evaluated based on a single fixed reconstruction under the same parameter settings. For 3DGS and the proposed NeRF-based scheme, the values are reported as mean ± standard deviation over three repeated trials. In each trial, PSNR, SSIM, and LPIPS were first averaged over the same test image set. These metrics were used to evaluate image-level rendering quality, while geometric accuracy was evaluated separately in Section 3.3 using relative radius error and circular fitting residuals. The arrows indicate the preferred direction of each metric: higher values are better for PSNR and SSIM, whereas lower values are better for LPIPS.

**Table 7:** Computational Cost and Output Applicability.

Stage/Method	Hardware	Runtime	Memory/Output
SfM, 35% retention	COLMAP	4.521 min	20,237 sparse points
SfM, 100% retention	COLMAP	197.044 min	29,210 sparse points
3DGS baseline	RTX 4070	31 min	Visual-quality comparison
Proposed NeRF scheme	RTX 4070	65 min	~9 GB GPU, ~22 GB RAM; 4,000,000 points
NeuS baseline	NVIDIA A100	169.67 min	Radius fitting not performed

Note: The SfM statistics are based on the NR-SNR retention-ratio experiment. The proposed NeRF scheme refers to the Nerfacto-based reconstruction pipeline used in this study. The NeuS baseline was evaluated under the tested configuration, and radius fitting was not performed because its output was not suitable for circular-section measurement.

**Table 8:** Comparison with a Representative SDF-Based Baseline.

Method	Training Config	Convergence	Applicability
Proposed NeRF scheme	100,000 iterations; RTX 4070/65 min	Reached scheduled training; output was geometrically usable	Applicable to circular-section fitting
NeuS baseline	100,000 iterations; NVIDIA A100/169.67 min	Final-stage loss remained relatively stable	Radius fitting was not performed

Note: The proposed NeRF scheme refers to the Nerfacto-based reconstruction pipeline used in this study. The NeuS baseline was evaluated under the tested configuration. Because its final output did not provide a shaft-wall geometry suitable for circular-section measurement, radius fitting was not performed. The absolute loss values of Nerfacto and NeuS are not directly compared because the two methods use different loss formulations. Detailed training logs are provided in the Supplementary File S1.

## Data Availability

The data presented in this study are available on request from the corresponding author. The data are not publicly available due to ongoing related research.

## Supplementary Materials

The following supporting information can be downloaded at: https://www.mdpi.com/article/10.3390/s26123847/s1, File S1: NeuS baseline training log for the SDF-based reconstruction experiment.
